# A Novel Test Method for Chloride Permeability of Ordinary Portland Cement Mortar Exposed to Salt Fog–Dry Cycles

**DOI:** 10.3390/ma19132772

**Published:** 2026-06-30

**Authors:** Qiwen Qiu, Denvid Lau

**Affiliations:** 1 School of Architecture and Civil Engineering, Huizhou University, Huizhou 516007, China; 2 Department of Architecture and Civil Engineering, City University of Hong Kong, Hong Kong 999077, China; denvid.lau@cityu.edu.hk

**Keywords:** durability, ordinary Portland cement mortar, test method, chloride permeability, salt fog–dry cycles, diffusion coefficient

## Abstract

**Highlights:**

- Chloride permeability of cement mortar under salt fog–dry cycles is investigated.
- An original salt fog–dry cycling test setup is proposed, established, and verified.
- A double-broken-line model is proposed to represent the steady-state chloride flux.
- Effective diffusion coefficient is proposed and determined using Fick’s first law.
- Effects of water/cement ratio and salt fog temperature on permeability are considered.

**Abstract:**

In this study, the stationary chloride permeability of ordinary Portland cement mortar exposed to salt fog–dry cycles is investigated. An original salt fog–dry cycling test setup, comprising an outer upstream tank and four inner downstream reservoirs, is established to explore chloride transport behavior through the bulk material. Steady-state chloride flux is discovered from the chloride profile, which exhibits a linear concentration gradient in both the convection and diffusion zones, following months of salt fog–dry cycles. Based on experimental observations, this study proposes a double-broken-line model to mathematically represent the chloride profile. An equivalent diffusion zone is then proposed by considering the deposited convection factor. Correspondingly, the diffusion coefficient of the equivalent diffusion zone is determined using Fick’s first law. Considering the effects of water-to-cement ratio and fog temperature, the stationary chloride permeabilities range from 0.283 × 10^−12^ m^2^/s to 0.674 × 10^−12^ m^2^/s, which are generally consistent with field data for concrete exposed to salt aerosols/dry environments. Although chloride permeability is temperature-dependent and, to some degree, affected by mixture proportion, the researchers recognize the aggregate/sample size effect on the variance of diffusivity values. Recommendations are drawn to upgrade the chloride transport scenario for a reliable evaluation of coarse-sand mortar and concrete.

## 1. Introduction

The ingress of atmospheric chlorides has increasingly been recognized as an aggressive factor that degrades coastal concrete infrastructure reinforced with steel. In marine atmosphere zones, chloride ions originating from airborne sea-salt aerosols can deposit on the surface of concrete structures and permeate into the porous concrete medium through complex transport mechanisms [1,2]. This causes depassivation of the embedded steel reinforcement, concrete distress, and an eventual reduction in the serviceability of reinforced structures [3,4,5]. Calculating the initiation time of metallic corrosion requires knowledge of the depth of chloride infiltration over time, which necessitates the characterization of the chloride transport coefficient of the bulk material under salt fog exposure conditions.

Many studies have aimed to understand the chloride transport behavior of ordinary Portland cement (OPC) concrete under salt spraying and drying cycles through actual field exposure or laboratory accelerated salt spraying tests [6,7,8]. Previous studies have reported the two-zone chloride profile comprising convection and diffusion regimes [9]. Some researchers are of the opinion that, in the convection zone, chloride ion penetration through unsaturated concrete is induced by cyclic moisture transport: capillary sorption in the wetting phase and water evaporation during drying [10]. Consequently, the chloride profile exhibits an increase in the ion concentration starting from the surface to an interior depth *x* at which the peak value *C_max_* is reached. Chloride movement beyond depth *x* is diffusion-driven under a chloride concentration gradient [9]. Many studies have been conducted to model the non-steady-state chloride diffusion flux [11,12], and the apparent nonstationary diffusion coefficient of this zone has been frequently determined using Fick’s second law [13]. Despite its widespread application, the accuracy of modeling chloride transport using Fick’s second law of diffusion is dictated by the complexity of the surface layer (e.g., convection zone) [10,14]. According to a study [13], *x* and *C_max_* are susceptible to uncertain factors, including time dependence under non-steady-state conditions. To predict chloride permeability more accurately, excessive efforts have been devoted to correlating *x* and *C_max_* with an age factor for different exposure conditions [13,15]. To the best of our knowledge, the nonstationary chloride diffusivities of OPC mortar and concrete range from 0.2 × 10^−12^ m^2^/s to 12.5 × 10^−12^ m^2^/s [16,17].

Previous laboratory and field studies have explored the time dependence of *C_max_* and recognized that this boundary condition tends to stabilize at an equilibrium over exposure time [17,18]. In the equilibrium condition, a constant chloride flux in the diffusion zone is eventually formed and can be used for a mathematical solution of the effective diffusion coefficient based on Fick’s first law. In this regard, the present study proposes a novel laboratory-based salt fog–dry cycling test designed to create a stationary ionic flux through the cement mortar. In addition, a theoretical model and physical equations of chloride transport through the equivalent diffusion zone are proposed. The apparatus is inspired by the conventional concentration gradient tests that induce one-dimensional steady-state mass transport [19]. Technically, the months-cured specimen is placed in an external upstream chamber, spraying salt fog, and an internal downstream reservoir filled with pure water. During salt fog–dry cycles, the chloride ion concentration in the downstream reservoir is recorded continuously to capture a stationary flow of chloride ions. A chloride profile with a depth interval of 500 μm is determined to validate the steady-state chloride flux. Compared to the nonstationary testing method, the steady-state salt fog–dry cycling test aims to achieve non-destructive testing of the chloride diffusion coefficient of cementitious materials. If *x* and *C_max_* are statistically analyzed using probabilistic methods, the chloride diffusion coefficient can be acquired by tracing the stationary flux of chloride ions passing over the sample. Compared with fitting the nonstationary data to Fick’s second law of diffusion, the stationary chloride permeation test avoids time-dependent material factors and improves the sensitivity and reproducibility of diffusion coefficient calculations. While it is worth examining steady-state chloride permeability, data on equilibrium chloride transport in earlier works concerning salt fog–dry cycles are rare. In addition, emphasis should be placed on revealing the impact of material and environmental dependence, namely, water-to-cement ratio and fog temperature, on the stationary transport properties. The proposed methodology is anticipated to be extended for determining the chloride diffusion coefficient of OPC concrete and blended concrete with different supplementary cementing materials.

## 2. Theory of Chloride Permeability of Mortar Under Salt Fog–Dry Cycles

### 2.1. Fick’s First Law of Diffusion

It has generally been accepted that the chloride ion penetration through cement-based materials obeys the Fickian process [20]. Regarding a unidirectional diffusion, Fick’s first law of diffusion connects the diffusive flux with the concentration gradient, as shown in Equation (1):

(1) $$J=-D\frac{dC}{dx}$$

where *J* represents the diffusion flux (mol/(m^2^·s)), *D* represents the ionic diffusivity (m^2^/s), and *C* represents the ionic concentration at distance *x* (mol/m^3^). This equation also implies that an increasing concentration gradient leads to the opposite direction of ionic flow. Concerning an OPC mortar, the steady-state diffusion flux induced by the concentration difference between the two ends of the bulk material can be defined by [21]:

(2) $$J=-D\frac{C_{1}-C_{2}}{h}$$

where *C*_1_ is the higher chloride concentration (mol/m^3^) at the upstream side, *C*_2_ is the chloride concentration (mol/m^3^) at the downstream reservoir of the test setup, and *h* is the thickness (m) of the sample. Using Equations (1) and (2) in the steady-state concentration gradient test requires that *C*_1_ is constant and a stationary ionic diffusion flux *J* is formed in the pore network of concrete.

### 2.2. Double-Broken-Line Model and Equivalent Diffusion Zone

**Figure 1:**
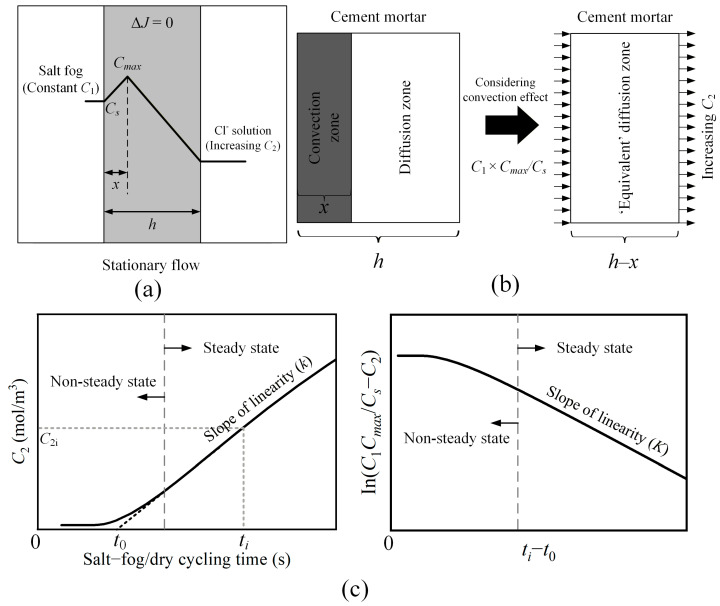
Theoretical representation of chloride transport kinetics of cement mortar subjected to steady-state salt fog–dry cycles: (**a**) double-broken-line model of chloride profile, (**b**) “equivalent” diffusion zone considering the convection deposition factor, and (**c**) graphical illustration of Equation (12) comprising the equivalent diffusion coefficient *D_eq_*.

In the salt fog–dry cycling test, *C*_1_ refers to the constant chloride concentration of salt aerosols, and *C*_2_ represents the chloride concentration gathered from the downstream reservoir. Compared with the conventional saltwater immersion test, the salt fog–dry cycling test can bring about the cyclic movement of moisture at the sub-surface layer of material due to capillary sorption and water evaporation (also known as ‘‘skin effect”). Consequently, the chloride profile exhibits a convection zone in which the chloride concentration increases from the surface chloride concentration (*C_s_*) to a maximum value (*C_max_*) at an interior depth *x* [10]. Although *C_max_* is a time-dependent parameter in the early cyclic period, it has been recognized that this concentration is prone to being stabilized over time [13,22,23]. Thus, in the proposed steady-state salt fog–dry cycling test, both *C_s_* and *C_max_* are assumed to reach a dynamic equilibrium after a certain number of environmental cycles. In this condition, the apparent flux *J* also becomes consistent over the convection and diffusion zones. Herein, the present study proposes a double-broken-line model, as graphically depicted in Figure 1a, to physically represent the chloride profile of OPC mortar under steady-state salt fog–dry cycles. In the convection domain, the chloride concentration increases linearly from *C_s_* to *C_max,_* at which an inflection point for the boundary between convection and diffusion zones is characterized. A deposited factor of *C_max_*/*C_s_* is thereby remarked. Beyond this inflection point, an “equivalent” diffusion zone is pointed out and illustrated in Figure 1b, and its diffusion coefficient is analyzed. As suggested in the literature [1,15], chloride ion penetration into concrete can be analytically characterized within the diffusion zone after rescaling the ionic concentration starting from the end of the convection zone. As the boundary condition of the “equivalent” diffusion zone, the source concentration becomes *C*_1_ × *C_max_*/*C_s_*, after considering the deposited factor of *C_max_*/*C_s_*. Accordingly, Equation (2) is modified as follows:

(3) $$J=-D_{eq}\frac{\frac{C_{1}C_{max}}{C_{s}}-C_{2}}{h-x}$$

where *D_eq_* denotes the equivalent diffusion coefficient (m^2^/s), *C_s_* is the chloride concentration (% wt. of mortar) of the mortar surface layer, and *C_max_* is the maximum chloride concentration (% wt. of mortar) in correspondence with distance *x*.

### 2.3. Theoretical Calculation of Equivalent Diffusion Coefficient

In the salt fog–dry cycling test, the steady-state chloride flux *J* is defined by the mass change in chloride ions per area and time. In other words, *J* is determined by linear regression of *C*_2_ versus time *t*, as follows:

(4) $$J=-\frac{V}{A}\frac{dC_{2}}{dt}$$

where *V* is the volume of the downstream reservoir (m^3^), and *A* is the cross-sectional transmission area of the test specimen (m^2^). Thus, Equations (3) and (4) yield the following:

(5) $$D_{eq}\frac{\frac{C_{1}C_{max}}{C_{s}}-C_{2}}{h-x}=\frac{V}{A}\frac{dC_{2}}{dt}$$

Equation (5) can be re-arranged in the form:

(6) $$\frac{D_{eq}A}{V(h-x)}dt=\frac{1}{\frac{C_{1}C_{max}}{C_{s}}-C_{2}}dC_{2}$$

Integrating both sides of this equation gives:

(7) $$\int \frac{D_{eq}A}{V(h-x)}dt=\frac{D_{eq}A}{V(h-x)}t$$

(8) $$\int \frac{1}{\frac{C_{1}C_{max}}{C_{s}}-C_{2}}dC_{2}=-In\left|\frac{C_{1}C_{max}}{C_{s}}-C_{2}\right|+C$$

In the stationary condition, *C*_2_ increases proportionally as the exposure time *t* increases. Linear regression of this part deduces the slope *k* and the projection of the abscissa (*t*_0_):

(9) $$C_{2}=k\left(t-t_{0}\right)$$

When Equations (7) and (8) are integrated, we obtain:

(10) $$\int_{t_{0}}^{t_{i}}\frac{D_{eq}A}{V(h-x)}dt=\frac{D_{eq}A}{V(h-x)}(t_{i}-t_{0})$$

(11) $$\int_{0}^{C_{2}}\frac{1}{\frac{C_{1}C_{max}}{C_{s}}-C_{2}}dC_{2}=In\left|\frac{C_{1}C_{max}}{C_{s}}\right|-In\left|\frac{C_{1}C_{max}}{C_{s}}-C_{2}\right|$$

Arranging the above two equations results in Equation (12):

(12) $$In\left|\frac{C_{1}C_{max}}{C_{s}}-C_{2}\right|=In\left|\frac{C_{1}C_{max}}{C_{s}}\right|-\frac{D_{eq}A}{V(h-x)}(t_{i}-t_{0})$$

Figure 1c presents the mathematical graph of this function. The linear slope of this function, namely *K* = *D_eq_A*/*V*(*h − x*), can be obtained by curve fitting and in turn the equivalent diffusion coefficient *D_eq_* is determined.

## 3. Experimental Procedures

### 3.1. Specimen Preparation

Ordinary Portland cement (OPC) mortars with three water-to-cement (*w*/*c*) ratios of 0.35, 0.40, and 0.50 were fabricated. All cement mortars had the sand-to-cement (*s*/*c*) ratio of 0.50. The cement had a strength grade of 42.5 (compressive strength ≥ 42.5 MPa at the 28-day curing), according to the specifications of the standard GB 175-2007 [24]. The chemical compositions of cement are listed in Table 1. The aggregate was the natural river sand with a fineness modulus of 2.7, complying with the standard GB/T 14684-2022 [25]. The size gradation of sand was listed here: 4% for 0–150 µm, 13% for 150–300 µm, 29% for 300–600 µm, 23% for 600–1180 µm, 14% for 1180–2360 µm, 13% for 2360–4750 µm, and 4% for 4,750 µm above. Three duplicate specimens were prepared for each mixture proportion, as detailed in Table 2. The first batch of nine prismatic specimens (i.e., M-N mixture) with dimensions of 10 mm × 10 mm × 7 mm was produced and tested at room temperature, varying with three *w*/*c* ratios of 0.35, 0.40, and 0.50. As the salt fog–dry cycling study also considered the environmental parameter of fog temperature, the secondary batch of nine cement mortars (i.e., M-H mixture) was prepared. All M-H mixture samples had the same *w*/*c* ratio of 0.35. To appropriately shorten the salt fog–dry cycling duration, researchers have attempted to reduce the thickness of mortar for M-H samples and made their dimensions 10 mm × 10 mm × 5 mm. Based on the size gradation of sand, the maximum aggregate size was around 4.75 mm, less than the thickness of the mortar sample. This is in agreement with the suggestion from previous chloride migration studies, implying that the thickness of the sample should be sufficient and larger than the maximum aggregate size to avoid the aggregate interface influence [26].

During the specimen preparation, the fresh mortar was formed by mixing ordinary Portland cement, river sand, and deionized water. After mixing, the fresh mortar was cast into cubic molds (12 mm × 12 mm × 12 mm) and placed on a vibrating plate until air bubbles ceased emerging on the surface. Once a prism was cast, the top surface was covered with wet burlap to avoid early shrinkage-induced cracks. Afterward, the specimens were subjected to 24 h initial setting, demolding, and the following three-month moist curing at room temperature for a sufficient hydration degree. It was assumed that the microstructure of well-cured cement mortar had minor or negligible change in the later salt fog–dry cycles. To reduce the effect of surface variability, the as-cast surface layer of the cured sample was removed and only the central portion of the prism was employed in the investigation. Specifically, the 2 mm outer parts of lateral section were first abrased via sandpaper, leaving the core area of 10 mm × 10 mm. Then, the front and back surface layers were similarly peeled, according to the type of mixture. The thicknesses of the M-N and M-H mixtures were set as 7 mm and 5 mm, respectively.

**Figure 2:**
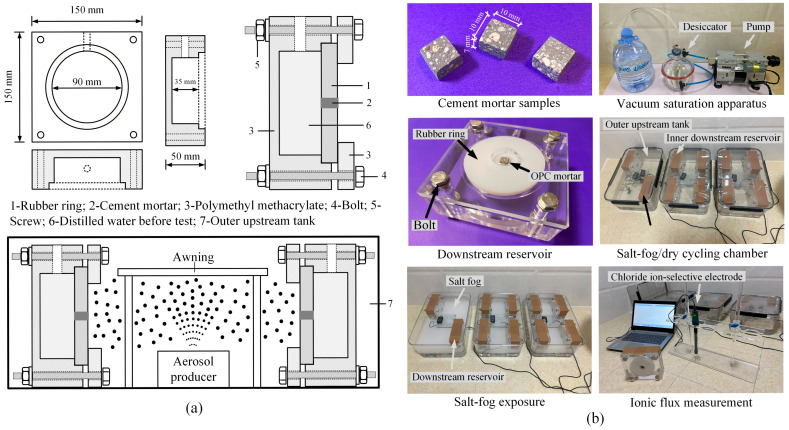
Salt fog–dry cycling test setup: (**a**) schematic diagram of cell reservoirs and salt spraying arrangement, and (**b**) photograph of test sample and experimental work.

The lateral specimen faces were waterproofed with an impermeable sealant, which was dedicated to a unidirectional chloride transport through the mortar specimen. All cement mortars were vacuum saturated with deionized water, following the standard ASTM C1202-22e1 [27]. For the first four hours, the samples were placed in a desiccator where the absolute pressure was reduced to 1000 Pa. After that, deionized water was drawn into the vacuum saturation apparatus by sub-atmospheric pressure to maintain sample immersion for another 18 h, as shown in Figure 2b. This procedure ensured that the samples were fully saturated at the beginning of the test.

### 3.2. Salt Fog–Dry Cycling Test

The proposed salt fog–dry cycling test setup was originally inspired by the classical two-compartment cell equipment. The apparatus comprised an outer upstream tank and inner downstream reservoirs. The lateral surfaces of the specimen were encased in a plastic rubber ring and sealed using a waterproof film, leaving the two ends exposed. The plastic ring was firmly screwed to a cylindrical single-sided downstream reservoir (90 mm in diameter and 35 mm in height), as schematically shown in Figure 2a. This configuration ensured that the front side of the sample was exposed to a salt fog–dry cyclic environment, and the back side was in contact with the downstream reservoir initially filled with deionized water. The cell reservoirs were then seated in the external salt fog–dry cycling chamber, namely, an upstream tank with dimensions of 427 mm × 300 mm × 158 mm. A four-liter sodium chloride solution with a mass concentration of 3.5% (approximately 620 mol/m^3^) was poured into the tank, after which the solution was completely renewed every week to maintain a constant concentration. An aerosol producer was positioned at the center of the external upstream chamber, which stimulated, sprayed, and settled the salt fog at a constant rate. Multi-concurrent tests could be performed as the external upstream chamber accommodated up to four downstream reservoirs. A video clip (Video S1) of salt fog spraying around the specimens during the test is available in the Supplementary Material of this article. The salt fog–dry alternation time ratio was set at 1:1 (12 h for salt fog spraying and the remaining 12 h for drying conditions). The salt fog temperature was measured regularly throughout the testing period, and the mean temperature was 33.98 °C. The top and bottom surfaces of the salt fog–dry cycling chamber were in contact with hot pads set at a constant temperature to consider the temperature factor of chloride permeability. The hot pad provided continuous heat to the chamber and maintained the salt fog temperature. According to the experimental goals, salt fog temperatures of 35 °C, 40 °C, and 50 °C were considered.

For mixture M-N representing the normal temperature case, the total salt fog–dry cycling duration or number of cycles was 434 days (cycles). For mixture M-H, representing the heated-temperature case, the test period was 280 days (cycles). Periodically, during the experiment, the chloride flux over the sample was recorded every two weeks. A 1 mL liquid sample from the inner downstream reservoir was collected using a pipette and dissolved 10 times in a graduated cylinder. A Go Direct^®^ chloride ion-selective electrode (ISE) was utilized to quantify the chloride concentration in the above aqueous samples, as described in Figure 2b. Before each time of regular testing, the ISE was carefully calibrated using the standard NaCl solutions at 1000 mg/L and 10 mg/L. To avoid the reading errors of the electrode owing to the ambient drying effect, the ISE was suspended and soaked in the aqueous sample for two minutes before recording the points of data. The concentration data (mg/L) were then collected for 1 min (sampling rate = 1 data point/second), and the averaged value was recorded. Notably, the calculation of chloride flux should consider the dilution effect of the liquid sample and convert the values back to represent *C*_2_ (chloride concentration at the inner downstream reservoir). The steady state of chloride ion movement was expected over a reasonable number of salt fog–dry cycles, thereby enabling the application of Fick’s first law to demarcate the diffusive flux driven by a concentration gradient.

### 3.3. Chloride Profile Measurement

After the salt fog–dry cycling test, the distribution of chloride ion concentration at different depths was plotted to shed light on the transport mechanism of chlorides. The measured chloride profile can also verify the theoretical double-broken-line model as mentioned above. The chloride ion concentration was represented by the water-soluble chloride content, which was expressed as a percentage by mass of the mortar. From the exposed surface, the prisms were manually sliced in steps of 500 μm each, checked by a vernier caliper, and powdered manually using a flat diamond saw. The 2 mm-thick residual part was not sliced but crushed and pulverized then. For each mortar slice, 30 mg of mortar powder samples were weighed by an analytical balance with a precision of 0.0001 g. Then, the samples were dissolved in 25 mL of water in a beaker for 5 min of boiling by means of a magnetic heating device. After standing 24 h, the mixture was filtered by suction, with a fine-texture filter paper, according to ASTM C1218/C1218M-20 [28]. The filtrate was adjusted to a neutral solution with a pH value of around 7 by adding a few drops of 0.1 mol/L nitric acid and hydrogen peroxide (3% solution). In addition, the volume of salt solution was adjusted to 40 mL before direct potentiometry. A Go Direct^®^ chloride ion-selective electrode (ISE) was used to determine the chloride concentration of the 40 mL solution. In the chloride profile, the chloride content was presented as the mass percentage of the water-soluble chlorides by weight of 30 mg of cement mortar.

## 4. Results and Discussion

### 4.1. Chloride Profile

In the present experiment, the chloride profile is measured to exhibit the chloride ion concentrations at different depths from the mortar surface toward the interior, revealing the kinetics of chloride ion transport under the salt fog–dry cycles. The free chloride ions were determined, as they are recognized to promote steel corrosion in concrete structures [16]. The chloride profiling was performed at 500 μm intervals to obtain a high resolution of *x* in which the convection and diffusion zones are precisely differentiated. Regarding the confidence level, the averaged values of chloride contents for three duplicate specimens in each mixture category were calculated. The corresponding deviations are listed in Tables S1 and S2 attached in the Supplementary Materials. Most of the deviations are less than 30% for the M-N mixture and less than 20% for the M-H mixture, which are generally acceptable for the measurement.

**Figure 3:**
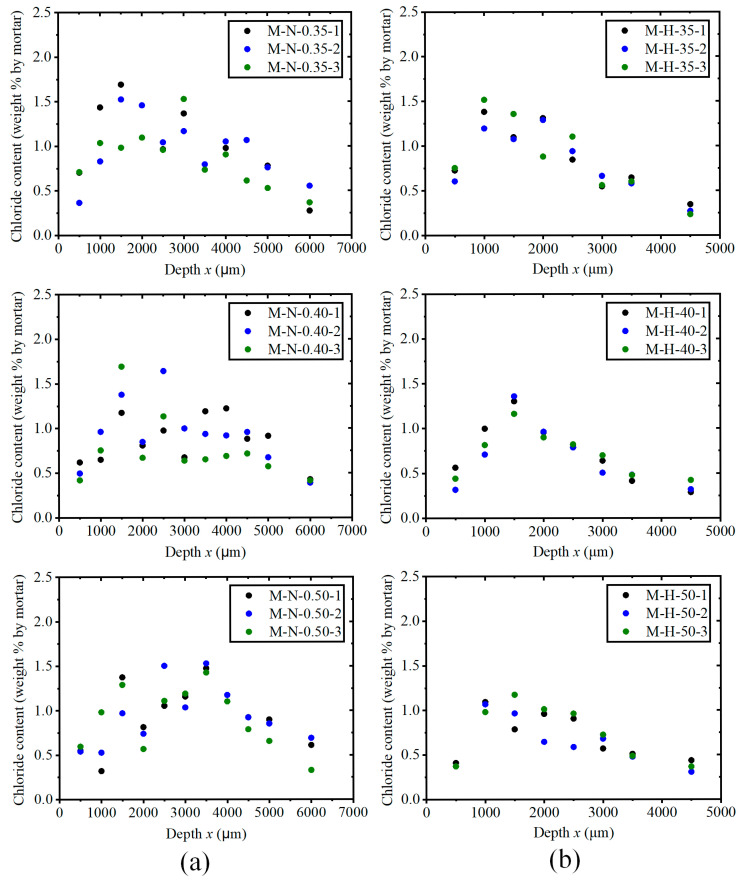
Chloride profile of OPC mortars under salt fog–dry cycles: (**a**) 7 mm-thick cement mortars with three *w*/*c* ratios of 0.35, 0.40, and 0.50, and (**b**) 5 mm-thick cement mortars under three temperature conditions of 35 °C, 40 °C, and 50 °C.

All types of specimens illustrate a peak in the two-zone chloride profile that is classified into convection and diffusion zones, as depicted in Figure 3. The peaks can be attributed to cyclic moisture transport in the cement mortar surface under drying and wetting cycles [29]. In the convection zone, a build-up of chloride concentration is observed at the first millimeters (1–3.5 mm) of the cement mortar. The convection zone is previously known as the “skin effect” owing to salt fog–dry cycles, and the length of this zone has been reported within 5 mm from most non-steady-state salt fog exposure studies [17,30,31,32]. Table 3 summarizes the values of *x* based on experimental observations of chloride profiles. The present exploration also demonstrates that the length of the convection zone depends on the material variant of the *w*/*c* ratio. An increasing *w*/*c* ratio deepens the convection zone, which may be attributed to an increased porous network for capillary sorption. In general, the higher the *w*/*c* ratio, the more chloride ions can penetrate deeper into the porous medium [29]. Similarly, a few previous salt fog–dry cycling investigations have discovered that the mixture proportion considerably affects the fundamental parameter of *x* in the two-zone chloride profile [1,17]. However, we did not figure out a remarkable change in *x* with the temperature rise. Table 3 also lists the calculated data of the ratio of maximum to surface chloride concentration (*C_max_*/*C_s_*), by averaging the values of three duplicate specimens for each mixture category. Concerning *C_max_*/*C_s_*, both *w*/*c* ratio and temperature show minor or negligible influence.

In Figure 3, the concentration gradients in both convection and diffusion zones are roughly linear, indicating the applicability of the double-broken-line model mentioned in Section 2. It has been argued that the mass transport of chloride ion tends to be equilibrious after certain salt fog–dry cycles, and *C_max_*/*C_s_* remains time-invariant [13,22,23]. In the early salt fog–dry exposure, the combined effects of capillary absorption and water evaporation bring up the concentration gradient between *C_max_* and *C_s_*. However, this reverse concentration gradient, in turn, enhances the back flow of diffusive ions from the interior *x* toward the exposed surface. The total effects of capillary sorption, water evaporation, and reverse ionic diffusion eventually lead to a net equilibrium flow of chloride ions through the convection zone. Simultaneously, the stationary flow of chloride ions occurs at the diffusion zone, complying with Fick’s first law of diffusion. The double-broken-line model can be utilized as a mathematical simplification of the chloride profile and a physical representation of constant apparent chloride flux across cement mortar. Thus, it is reasonable for Fick’s first law to quantify the equivalent diffusion coefficient of the diffusion zone once the steady-state chloride flux is determined.

### 4.2. Steady-State Chloride Flux

**Figure 4:**
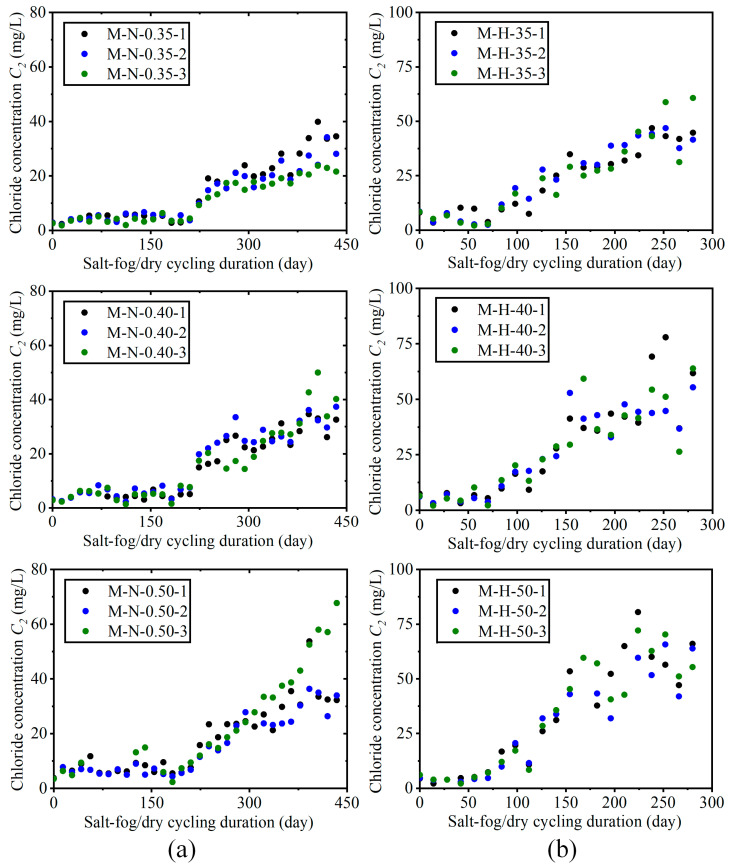
Time-varying chloride concentration *C*_2_ at downstream reservoir: (**a**) *w*/*c* ratios of 0.35, 0.40, and 0.50, and (**b**) temperature variations of 35 °C, 40 °C, and 50 °C.

During the salt fog–dry cycling test, the time-varying free chloride concentration *C*_2_ was measured, recorded, and plotted in Figure 4. In the first few months, only some slight fluctuations in the values of concentrations are observed. After successive months of salt fog–dry cycles, *C*_2_ exhibits a linear increase from 20 to 50 mg/L. The linearity indicates the outcome of steady-state chloride flux through the cement mortar. This phenomenon is tallied with the observation of a linear concentration gradient of the diffusion zone in Figure 3. In addition, *C*_2_ exhibits a larger slope for a high *w*/*c* ratio of 0.50. It is generally accepted that the *w*/*c* ratio has appreciable impacts on the porosity of hardened cement mortar and the effective diffusion of chloride in the porous medium [33,34]. This influential factor has been found in the cases of salt fog exposure and chloride movement [35]. It can also be observed from Figure 4b that the chloride ion flux rises by increasing the temperature from 35 °C to 50 °C. In addition, it takes a shorter period of salt fog–dry cycling for an observation of steady-state chloride flux for the M-H mixtures, compared to the M-N mixtures. The temperature-dependent behavior of salt fog-related chloride transport agrees with those findings from previous studies [3,36]. During mass transport, an increasing temperature results in a higher molecular velocity of diffusing species and more intensive thermal vibration of chloride ions physically bound to the cement hydrate surface [37]. The above observations demonstrate the occurrence of a steady-state flux of chloride ions passing through the bulk material, indicating a new test approach scope for characterizing chloride permeability.

### 4.3. Equivalent Diffusion Coefficient

**Figure 5:**
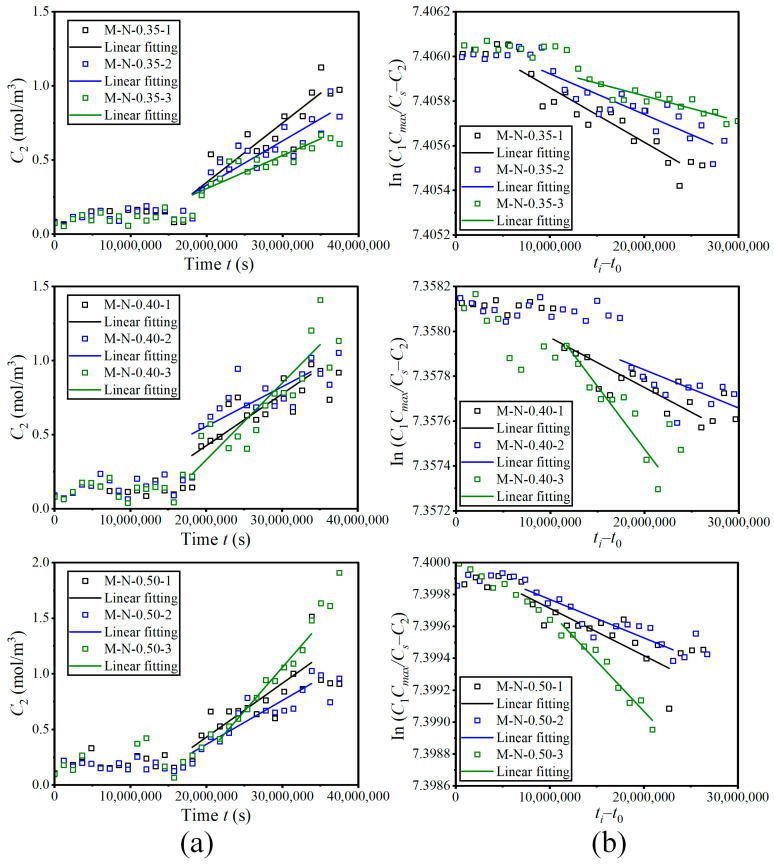
Chloride ion concentration over time for the M-N mortar mixes: (**a**) *C*_2_–*t* relation and (**b**) relationship between In(*C*_1_*C_max_/C_s_*–*C*_2_) and *t_i_*–*t*_0_.

**Figure 6:**
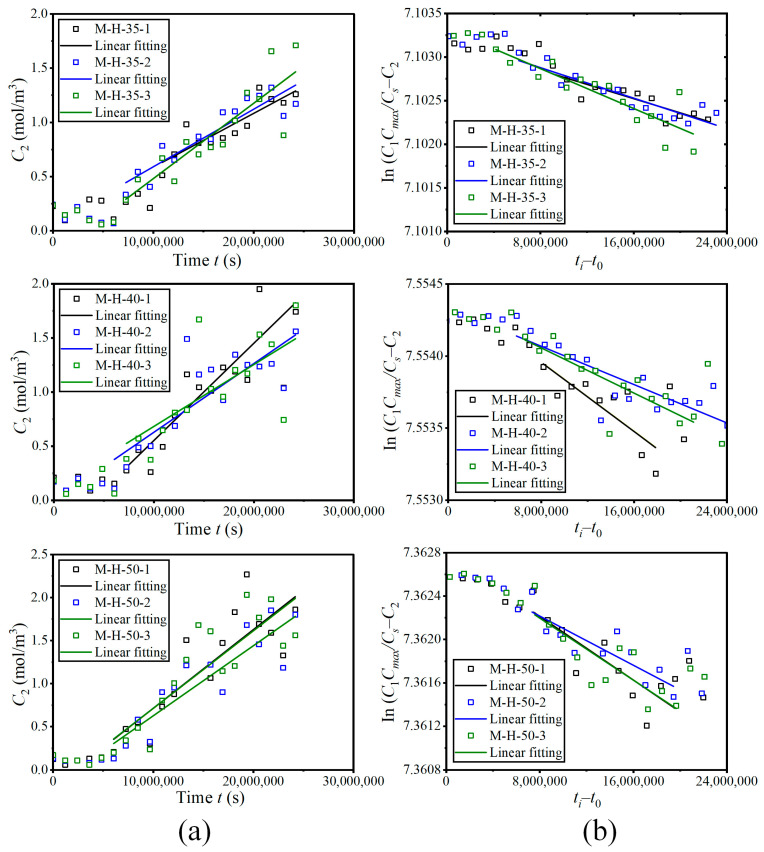
Chloride ion concentration over time for the M-H mixes: (**a**) *C*_2_–*t* relation and (**b**) relationship between In(*C*_1_*C_max_/C_s_*–*C*_2_) and *t_i_*–*t*_0_.

Considering the skin effect, the equivalent diffusion zone in the stationary chloride profile is modeled using Fick’s first law. As shown in Figure 5a and Figure 6a, linear regression is used to fit the time-varying chloride concentration *C*_2_ recorded at the downstream reservoir, resulting in a linear slope *k* and a time parameter *t*_0_. The experimental data and the curve-fitting results based on Equation (12) are presented in Figure 5b and Figure 6b, respectively. Equation (12) provides a reasonably good approximation of the linearity of the experimental relationship.

**Figure 7:**
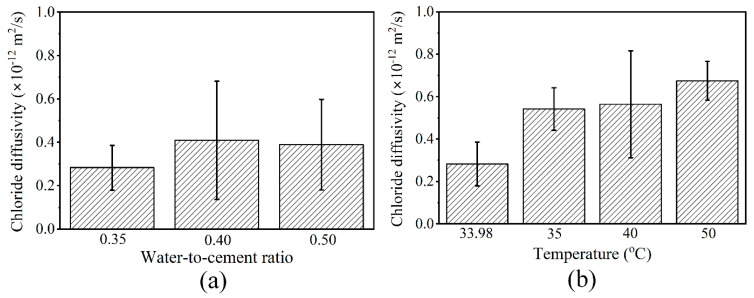
Influence of water-to-cement ratio and salt fog temperature on equivalent chloride diffusivity (*D_eq_*) of cement mortar subjected to salt fog–dry cycles: (**a**) *D_eq_* versus water-to-cement ratio and (**b**) *D_eq_* versus salt fog temperature.

From the calculations using Equation (13), the steady-state equivalent diffusion coefficients (*D_eq_*) of OPC mortars are determined and summarized in Table 4 and Table 5. The averaged diffusivities are in the range of 0.283 × 10^−12^ to 0.674 × 10^−12^ m^2^/s, as presented in Figure 7. The values have a similar magnitude to the results (0.2 × 10^−12^ to 1.2 × 10^−12^ m^2^/s) for field-cast OPC concretes exposed to airborne salts [22,30,31,38]. However, the equivalent diffusion coefficients seem lower than those obtained by steady-state natural diffusion tests (0.887 × 10^−12^ to 1.043 × 10^−12^ m^2^/s) and steady-state migration tests (0.74 × 10^−12^ to 6.26 × 10^−12^ m^2^/s) [39]. It has been pointed out that the external electrical current can cause the electro-coupling effect on the multi-species ions in the pore solution, and, as such, its chloride diffusion behavior is significantly different. In addition, the calculations of steady-state diffusion coefficients consider the duration of the dry state in which the cement mortar is unsaturated, with decreasing connectivity of the pore solution. The diffusion coefficients can be reduced because the ionic diffusion requires the continuous existence of water in the pore network [6,40]. Meanwhile, during the salt fog–dry cycles, the salt fog is inconsistently supplied to the surface of the mortar, which further limits the kinetics of chloride diffusion. The effect of material size is also an influencing factor; for example, the millimeter-sized specimen could have a better fineness of pore structure, and a low permeability may manifest.

(13) $$D_{eq}=\frac{KV(h-x)}{A}$$

where *K* denotes the linear slope of the curve fitting obtained using Equation (12).

The equivalent chloride diffusivity of cement mortar seems smaller, considering the high compactness of the bulk material. As displayed in Figure 7a, the averaged diffusivity of cement mortar with a *w*/*c* ratio of 0.35 is apparently smaller than that with higher *w*/*c* ratios of 0.40 and 0.50. Nevertheless, there appears to be little difference between the diffusivities with the *w*/*c* ratios of 0.40 and 0.50. More variance of diffusivity values is found for mortars with high *w*/*c* ratios. The deviation can be associated with the finite size effect of the sample, where the ionic transport of cement mortar is more susceptible to the location of sand aggregate. To reduce this error, future investigation efforts should be devoted to modifying and dilating the lateral boundary of the specimen, for example, 25 mm × 25 mm for coarse-sand mortar and 50 mm × 50 mm for concrete.

The chloride transport behavior is also temperature-dependent. As mentioned above, elevated fog temperature leads to earlier formation of steady-state chloride flux. Although the total amount of environmental cycles between M-N and M-H mixtures is different in the test, *x* and *C_max_*/*C_s_* are assumed to be stabilized, and, correspondingly, the stationary diffusion coefficients determined using Fick’s first law are comparable between them. Increasing the salt fog temperature brings up the equivalent diffusion coefficients, but the increasing mode does not seem to be the Arrhenius type that has been previously recognized in saturated concretes [41,42,43]. Considering that the serviceability of concrete structures can degrade in hot marine atmosphere environments, the temperature should be deemed a crucial factor in the durability analysis of concrete. For an effective performance-based design, the prescriptive rules or code requirements, such as minimum concrete cover depth, should be established in accordance with the temperature.

### 4.4. Further Discussions

In the above discussions, it is experimentally confirmed that the salt fog–dry cycles lead to a convection-diffusion profile that can be mathematically described by a double-broken-line model. Based on this model, the equivalent diffusion coefficient *D_eq_* of the diffusion zone is quantitatively determined. Additionally, the double-broken-line model indicates the linear concentration gradient and the constant flux in the convection zone. Thus, a governing equation of chloride transport for the convection zone can also be suggested, analogous to the form of Fick’s first law, as described in Equation (14). A convection coefficient *K* is derived to correlate the apparent stationary chloride flux with the concentration gradient, which is the combined result of cyclic moisture movement and reverse ionic diffusion.

(14) $$J_{a}=K\frac{C_{max}-C_{s}}{x}$$

where *J_a_* denotes the apparent chloride flux. Based on the definition of chloride ionic flux mentioned above, Equations (4) and (14) can be expressed as

(15) $$K\frac{C_{max}-C_{s}}{x}=\frac{V}{A}\frac{dC_{2}}{dt}$$

Integrating Equation (15) can generate

(16) $$\frac{C_{2}}{C_{max}-C_{s}}=\frac{KA}{Vx}(t_{i}-t_{0})$$

Equation (16) gives the mathematical form that is applicable in the salt fog–dry cycling test to estimate the transport properties of the outer skin. The convection coefficient *K* indicates the rate of accumulation of chloride ions in this zone. By combining the convection and diffusion coefficients, we further conceive an extended model that accounts for the overall transport properties in both zones. The model is inspired and conceptualized from the double-capacitance layer of Goüy–Chapman [44,45]. It can be visualized as a series circuit combination of a “charging capacitor” and a “discharging capacitor”. The convection zone acts as the “charging capacitor” for depositing ions, which contributes to a concentration gradient and drives the ionic transport. Meanwhile, the outer boundary of this zone is considered to carry the ions by diffusion. The diffusion zone plays the role of the “discharging capacitor”, which dilutes the ions. Based on the principle of calculating the capacitance of the combined capacitors and the thickness ratio of each zone, an overall chloride permeability expression that considers the convection and diffusion mechanisms is given by Equation (17):

(17) $$P=\frac{1}{\frac{1}{h}(\frac{x}{K}+\frac{h-x}{D_{eq}})}$$

where *P* denotes the overall chloride permeability (m^2^/s) of cement mortar under salt fog–dry cycles. *P* can also be interpreted as an extended diffusion coefficient or harmonized diffusivity, which highlights a more generalized indicator for previewing the expectancy of material transport behavior. When *x* = 0, the chloride transport is only driven by the diffusion zone. This is totally consistent with the saltwater-submerged scenario. In this case, *P* is represented by a diffusion coefficient. When *x* = *h*, diffusion no longer plays a role in the entry of chloride ions; thus, *P* is simplified as a convection coefficient. When 0 < *x* < *h*, both convection and diffusion zones contribute to the ingress of chloride ions.

### 4.5. Future Study

The present original work focuses on the salt fog–dry cycling experiment in which the steady-state chloride diffusion coefficient of OPC mortar, as a key transport coefficient for durability analysis of a conventional reinforced concrete structure, can be evaluated. It is also noteworthy that the future study is directed toward the feasibility of testing the transport properties of concrete using this proposed test method. Testing concrete materials requires a larger size (thickness ≥ 15 mm) of specimen, considering the presence of coarse aggregate. It will require a long salt fog–dry cycling duration to observe the steady-state chloride flux. Thus, future research should focus on the automation of the salt fog–dry cyclic process for an efficient evaluation. Furthermore, the application of the novel salt fog–dry cycling test can be extended to high-performance concrete and polymer-matrix composites that exhibit more multi-scale structural characteristics and environmental benefits than OPC concrete [46,47,48,49]. Future studies are also meaningful to determine the stationary chloride diffusion coefficient of blended cement mortar and concrete with waste products, recycled aggregate, nanoparticles, etc.

## 5. Conclusions

The original research proposes and investigates a test method for chloride permeability of OPC mortar exposed to salt fog–dry cycles, offering insights into durability prediction, safety design, and service-life analysis of partially saturated cementitious materials used in marine atmosphere environments. An equivalent diffusion coefficient of OPC mortar under salt fog–dry cycles is theoretically proposed and experimentally determined. During the salt fog–dry cycling test, the steady-state chloride ion flux through the specimen is explored and recorded, as the linear increase in concentration in the downstream reservoir is found. The chloride profile also confirms a linear concentration gradient in the diffusion zone, whereas the convection zone shows an opposite chloride concentration trend. A double-broken-line model is proposed to represent the chloride profile under salt fog–dry cycles. The study also conceives an equivalent steady-state diffusion zone by considering the deposition effect in the convection zone. A solution based on Fick’s first law is derived to determine the diffusion coefficient of the equivalent diffusion zone, ranging from 0.283 × 10^−12^ m^2^/s to 0.674 × 10^−12^ m^2^/s. Although the *w*/*c* ratio and salt fog temperature, to some degree, are found to influence the chloride permeability of mortar subjected to salt fog environments, an obvious variance of diffusivity values can be induced by sand aggregate due to the small sample size. Future efforts should be devoted to dilating the lateral boundary of the specimen to reduce this error, especially to evaluate coarse-sand mortar and concrete.

## Figures and Tables

**Table 1:** Chemical compositions of OPC used in the experiment (mass percentages of oxides).

Composition	CaO	SiO_2_	Al_2_O_3_	Fe_2_O_3_	MgO	SO_3_
Value (%)	66.7	22.03	6.8	4.20	3.22	2.32

**Table 2:** Mixture ID and mixture proportion of OPC mortars.

Mixture ID	*w*/*c* Ratio	*s*/*c* Ratio	Mixture ID	*w*/*c* Ratio	*s*/*c* Ratio
M-N-0.35-1	0.35	0.50	M-H-35-1	0.35	0.50
M-N-0.35-2			M-H-35-2		
M-N-0.35-3			M-H-35-3		
M-N-0.40-1	0.40	0.50	M-H-40-1	0.35	0.50
M-N-0.40-2			M-H-40-2		
M-N-0.40-3			M-H-40-3		
M-N-0.50-1	0.50	0.50	M-H-50-1	0.35	0.50
M-N-0.50-2			M-H-50-2		
M-N-0.50-3			M-H-50-3		

Note: In the mixture ID, “M” denotes mortar; “N” denotes normal temperature; “H” denotes heated temperature; “0.35”, “0.40”, and “0.50” refer to the values of *w*/*c* ratio; “35”, “40”, and “50” refer to the fog temperatures (°C) during the salt spraying; “1”, “2”, and “3” denote the series number of each mixture category.

**Table 3:** Effects of the *w*/*c* ratio and temperature on *x* and *C_max_*/*C_s_* for OPC mortars under salt fog–dry cycles.

Parameter	*x* (mm)						*C_max_*/*C_s_*					
Factor	*w*/*c* ratio			*T* (°C)			*w*/*c* ratio			*T* (°C)		
	0.35	0.40	0.50	35	40	50	0.35	0.40	0.50	35	40	50
Value	1.5	2.5	3.5	1.0	1.5	1.5	2.655	2.531	2.639	1.961	3.079	2.542

**Table 4:** Equivalent chloride diffusivity (*D_eq_*) of cement mortars with different *w*/*c* ratios.

*w*/*c* Ratio	Sample No.	Diffusion Coefficient (m^2^/s)
0.35	M-N-0.35-1	0.384 × 10^−12^
	M-N-0.35-2	0.286 × 10^−12^
	M-N-0.35-3	0.178 × 10^−12^
0.40	M-N-0.40-1	0.286 × 10^−12^
	M-N-0.40-2	0.219 × 10^−12^
	M-N-0.40-3	0.721 × 10^−12^
0.50	M-N-0.50-1	0.297 × 10^−12^
	M-N-0.50-2	0.242 × 10^−12^
	M-N-0.50-3	0.628 × 10^−12^

**Table 5:** Equivalent chloride diffusivity (*D_eq_*) of cement mortars subjected to different temperature conditions.

Temperature	Sample No.	Diffusion Coefficient (m^2^/s)
35 °C	M-H-35-1	0.468 × 10^−12^
	M-H-35-2	0.501 × 10^−12^
	M-H-35-3	0.655 × 10^−12^
40 °C	M-H-40-1	0.852 × 10^−12^
	M-H-40-2	0.385 × 10^−12^
	M-H-40-3	0.454 × 10^−12^
50 °C	M-H-50-1	0.737 × 10^−12^
	M-H-50-2	0.569 × 10^−12^
	M-H-50-3	0.717 × 10^−12^

## Data Availability

The original contributions presented in this study are included in the article/Supplementary Materials. Further inquiries can be directed to the corresponding author.

## Supplementary Materials

The following supporting information can be downloaded at: https://www.mdpi.com/article/10.3390/ma19132772/s1, Table S1: Averaged chloride content with depth *x* and the measurement deviation for M-N mixture; Table S2: Averaged chloride content with depth *x* and the measurement deviation for M-H mixture; Video S1: Video_Salt fog exposure; Raw data.zip.
