# Supplementary material for: A Novel Test Method for Chloride Permeability of Ordinary Portland Cement Mortar Exposed to Salt Fog–Dry Cycles
Source: Materials (Basel). 2026 Jun 30;19(13):2772. doi: 10.3390/ma19132772 (PMC13362727; doi:10.3390/ma19132772)
Supplement: Supplementary file 1 [file materials-19-02772-s001.zip › Tables S1 and S2_Averaged chloride content with depth x and the measurement deviation.pdf]

Table S1. Averaged chloride content with depth *x* and the measurement deviation for M-N mixture.

**Depth (μm)**	**Mixture catagory**					
	M-N-0.35		M-N-0.40		M-N-0.50	
	Average	Deviation (%)	Average	Deviation (%)	Average	Deviation (%)
500	0.593	19.740	0.512	10.11	0.562	2.981
1000	1.101	30.790	0.789	15.930	0.610	33.885
1500	1.400	37.017	1.415	26.095	1.212	21.270
2000	1.278	25.650	0.776	9.300	0.708	12.634
2500	0.989	4.694	1.251	34.828	1.223	24.481
3000	1.355	18.071	0.771	19.888	1.128	8.147
3500	0.765	4.255	0.927	26.934	1.478	5.116
4000	0.979	7.325	0.944	26.743	1.139	5.125
4500	0.840	32.067	0.853	12.350	0.879	7.835
5000	0.689	13.993	0.720	17.543	0.803	12.864
6000	0.398	14.216	0.410	1.935	0.544	19.003

Table S2. Averaged chloride content with depth *x* and the measurement deviation for M-H mixture.

**Depth (μm)**	**Mixture catagory**					
	M-H-35		M-H-40		M-H-50	
	Average	Deviation (%)	Average	Deviation (%)	Average	Deviation (%)
500	0.696	7.972	0.440	12.312	0.371	2.746
1000	1.366	16.096	0.841	14.606	0.982	5.882
1500	1.178	15.644	1.275	10.084	1.175	19.430
2000	1.161	24.236	0.940	3.457	1.013	19.870
2500	0.965	12.995	0.799	1.907	0.963	20.262
3000	0.591	6.408	0.615	9.923	0.725	7.999
3500	0.610	3.360	0.458	3.878	0.487	1.663
4500	0.285	5.665	0.343	7.073	0.366	6.478
